# Polypharmacological Profiles Underlying the Antitumor Property of *Salvia miltiorrhiza* Root (Danshen) Interfering with NOX-Dependent Neutrophil Extracellular Traps

**DOI:** 10.1155/2018/4908328

**Published:** 2018-08-19

**Authors:** Li Tao, Min Xu, Xiaojun Dai, Tengyang Ni, Dan Li, Feng Jin, Haibo Wang, Lide Tao, Bo Pan, James R. Woodgett, Yayun Qian, Yanqing Liu

**Affiliations:** ^1^College of Medicine, Yangzhou University, Yangzhou, Jiangsu 225001, China; ^2^The State Administration of Traditional Chinese Medicine Key Laboratory of Toxic Pathogens-Based Therapeutic Approaches of Gastric Cancer, Yangzhou University, Yangzhou, Jiangsu 225009, China; ^3^Yangzhou Hospital of Chinese Medicine, Yangzhou, Jiangsu 225009, China; ^4^Affiliated Hospital of Yangzhou University, Yangzhou, Jiangsu 225001, China; ^5^Lunenfeld-Tanenbaum Research Institute, Sinai Health System, Toronto, ON, Canada M5G 1X5; ^6^Department of Medical Biophysics, University of Toronto, Toronto, ON, Canada M5G 1X5

## Abstract

Danshen, the dried root of *Salvia miltiorrhiza*, one of the most investigated medicinal plants with well-defined phytochemical constituents, has shown prominent clinical outcomes for antioxidant, anti-inflammatory, and anticoagulant activities to attain vascular protection and additional benefits for cancer therapy. More recently, activation of neutrophil and excessive formation of neutrophil extracellular traps (NETs) have been observed in pathological conditions of metastatic cancers; thus, we hypothesized that suppression of NETs could account for an essential cellular event underlying Danshen-mediated reduction of the incidence of metastasis. Using an experimental pulmonary metastases model of red fluorescent protein- (RFP-) labeled gastric cancer cells in combination with macroscopic ex vivo live-imaging system, our data indicated that Danshen impaired the fluorescent intensity and quantity of metastatic nodules. Moreover, Danshen could prevent neutrophil trafficking to the metastatic sites with decreased plasma levels of neutrophil elastase (NE) and procoagulant potential featured by fibrinogen. We further established phorbol 12-myristate 13-acetate- (PMA-) induced NET formation of human neutrophils and screened representative active compounds derived from the hydrophilic and hydrophobic fractions of Danshen using qualitative and quantitative methods. As a result, we found that salvianolic acid B (Sal B) and 15,16-dihydrotanshinone I (DHT I) exhibited superior inhibitory activities on NET formation and significantly attenuated the levels of citrullinated histone H3 (citH3), a biomarker for NET formation. Multitarget biochemical assays demonstrated that Sal B and DHT I distinctly modulated the enzymatic cascade involved in NET formation. Sal B and DHT I could disrupt NET formation at the earlier stage by blocking the activities of myeloperoxidase (MPO) and NADPH oxidase (NOX), respectively. Lastly, combining treatment of Sal B and DHT I under subED_50_ doses displayed remarkable synergism effect on NET inhibition. Altogether, these data provide insight into how promiscuous compounds from herbal medicine can be effectively targeted NETs towards hematogenous metastasis of certain tumors.

## 1. Introduction

Chinese materia medica (CMM) holds great potential to improve people's health and wellness and needs rigorous scientific data and deeper knowledge to demonstrate its pharmacological significance at a molecular level [1]. Danshen, the dried root of *Salvia miltiorrhiza*, the first herbal remedy successfully passed phase II clinical trials under the Food and Drug Administration (FDA) in USA, has been intensively studied for its protective role against cardiovascular diseases [2–4]. Traditionally, the herb is featured by blood activating and stasis resolving (BASR) function as an allopathic regimen to treat thrombotic disorders. Notably, Danshen is a highly ranked CMM represented in clinical prescriptions to treat advanced tumor patients with blood stasis syndrome [5]. Indeed, a body of evidence has delineated procoagulant mechanisms in the context of metastatic cancers [6], and the potential clinical impact of Danshen on malignant disease is a novel matter of scientific debate currently [2, 7, 8].

In addition to tumor-infiltrating neutrophils that constitute major tumor-promoting properties in the tumor microenvironment, a newfound appreciation for neutrophils on venous thrombosis has been summarized [9]. Neutrophil extracellular traps (NETs) through externalization of their nuclear DNA provide a scaffold that sequesters circulating tumor cells essential for successful metastasis. NET formation that can be suicidal to neutrophils, or termed as “NETosis,” has been previously recognized as innate immunity fighting extracellular pathogens and also implicated in hematogenous metastasis mediated by tumor-associated thrombosis [10, 11]. This is particularly of high interest for abrogating metastasis by targeting NETosis [12, 13].

Typically, an oxidative burst of reactive oxygen species (ROS) is predominantly generated by NADPH oxidase (NOX) as an early response to phagocytosis-mediated microbicidal activity of neutrophils during NETosis. This further requires protein-arginine deiminase 4 (PAD4) to catalyze the conversion of peptidyl-arginine to peptidyl-citrulline on histone tails for intracellular chromatin decondensation [14]. Studies have revealed that inhibition of PAD4 activity using genetic knockout of PAD4 or chemical inhibitor of PAD4 is sufficient to disrupt NET formation [15, 16]. Specifically, citrullinated histone H3- (citH3-) positive neutrophils indicate a prothrombotic state in tumor patients [17, 18]. During this process, abundant granular proteases and peroxidases including neutrophil elastase (NE) and myeloperoxidase (MPO) translocate to the nucleus to degrade histones and promote chromatin decondensation followed by nuclear membrane rupture that allows chromatin to mix with granule contents. Furthermore, MPO reacts with hydrogen peroxide to produce hypochloric acid (HOCl) primed for NET release [19, 20]. NET-dissolving drugs like heparin and DNase I could substantially reduce metastases in mice [21]. Therefore, there exists a complicated enzymatic cascade consisting of NOX, PAD4, NE, MPO, and so on, which plays a pivotal role in sequentially triggering NETosis [22].

The systematic role of neutrophils in metastasis which we believe might offer a novel perspective for BASR medicine in cancer therapy. The primary bioactive compounds in Danshen include the hydrophilic caffeic acid derivatives and the lipophilic diterpenoid tanshinones. Representative caffeic acid derivatives including caffeic acid (CA), danshensu (DSS or 3,4-dihydroxyphenyllactic acid), rosmarinic acid (RA, a condensation product from one molecule of CA and one molecule of DSS, and salvianolic acid B (Sal B, a condensation product from one molecule of CA and three molecules of DSS), while tanshinone I (Tan I), 15,16-dihydrotanshinone I (DHT I), tanshinone IIA (Tan IIA), and cryptotanshinone (CPT) are the most abundant compounds among diterpenoid tanshinones [23]. Previously, we have identified that the phenolic acids from BASR medicine including CA, DSS, RA, and Sal B could inhibit cyclooxygenase-2 activity and reduce prostaglandin E_2_ production in tumor cells [24]. There are diverse types of commercially available Chinese patent drugs prepared from Danshen extract. In the present study, we utilized intravenous Danshen injection (DSI) to investigate the antimetastatic properties of Danshen in an experimental pulmonary metastasis mice model *in vivo* and screened effective compounds derived from Danshen on NET formation *in vitro*. The pharmacological mechanism was further focused on the enzymatic functions involved in NETosis. Thus, our outcomes may further advance the scientific significance of BASR medicine in tumor therapy and the relevance of conventional BASR functions and anticancer properties in future clinical application.

## 2. Materials and Methods

### 2.1. Cell Cultures

The human GC cell lines BGC-823 (Cell Resource Center of Chinese Academy of Sciences, Shanghai, China, Cat number TCHu 11) were maintained in RPMI 1640 media containing 10% FBS. The Red fluorescent protein- (RFP-) labeled BGC-823 cells were established by transduction of RFP-expression lentiviral (Lv) containing a puromycin-resistance gene for selection of stable cells under CMV promoter (Applied Biological Materials Inc. or ABM, Cat number LV076). BGC-823-RFP cells were maintained in complete RPMI 1640 media with 2 *μ*g/mL of puromycin. Cultures were negative of mycoplasma after testing with EZ-PCR mycoplasma test kit (Biological Industries, Cat number 20-700-20).

### 2.2. Chemicals

Intravenous Danshen injection (DSI) was purchased from Sunnyhope Pharmaceutical Co. Ltd. (Sichuan, China, approval license number: Z51021303) and prepared with the dried roots of the plant at the concentration of 1.5 g/mL aqueous extract following national drug standards (number WS3-B-3766-98-2011). The Certificate of Analysis (COA) for the product specification (Lot. number: 1704114) is shown in Supplementary Document 1. The contents of total phenolic acids, danshensu sodium salt, protocatechuic aldehyde, rosmarinic acid, and salvianolic acid B were 8.2, 0.97, 0.30, 0.37, and 0.48 mg/mL, respectively. Paclitaxel injection was purchased from Angtze River Pharmaceutical (Group) Co. Ltd. (Taizhou, China, approval license number: H20058719). Purified compounds (HPLC ≥ 98%) from the plant, including Tan I (CAS number 568-73-0, Cat number ST8010), Tan IIA (CAS number 568-72-9, Cat number ST8020), DHT I (CAS number 87205-99-0, Cat number SD8290), CPT (CAS number 35825-57-1, Cat number SC8640), DSS (CAS number 76822-21-4, Cat number SD8030), CA (CAS number 331-39-5, Cat number SC8010), RA (CAS number 20283-92-5, Cat number SR8190), and Sal B (CAS number 115939-25-8, Cat number SS8100) were obtained from Solarbio Science & Technology Co. Ltd. (Beijing, China). All the chemical information has been listed in Supplementary Table 1.

### 2.3. Animals and Experimental Pulmonary Metastasis Model

Animal research and corresponding animal utilization protocol (AUP) were approved by the Animal Care and Use Committee of College of Medicine, Yangzhou University. Studies with animals are described in compliance with the ARRIVE guidelines for reporting experiments involving animals [25]. Five- to six-week-old female BALB/c athymic nude mice were obtained and housed by the animal facility of Comparative Medicine Center, Yangzhou University, under specific pathogen-free (SPF) condition. 5 × 10^6^ cells/mL of BGC-823-RFP cells suspended in 200 *μ*L of PBS were injected into the tail vein of each nude mouse. After inoculation, mice were randomized into three groups (*n* = 8 per group) according to their body weight. From the next day, mice were intraperitoneally (i.p.) injected with paclitaxel (10 mg/kg, every 3 days for a total of 9 injections on days 1, 4, 7, 10, 13, 16, 19, 22, and 25) or DSI (780 mg/kg, q.d. for a total 28 injections). Control animals were treated with normal saline (N.S.). After four weeks, mice were assigned to isoflurane anesthesia. Blood was rapidly withdrawn from each mouse by cardiac puncture and collected into 1.5 mL heparin-coated Eppendorf tubes. Immediately, mice were sacrificed by carbon dioxide asphyxiation, and then lung tissues were harvested and rinsed with PBS. Pulmonary metastases were visualized using IVIS™ live-imaging system (IVIS Lumina III, PerkinElmer) at Ex/Em: 600/710 nm. Quantification of metastatic foci was performed by measuring epi-fluorescence intensity of the whole lung region as “regions of interest” (ROI). Lungs were fixed with formaldehyde solution for 24 hours and analyzed for histological lung colonization by hematoxylin and eosin (H&E) staining.

### 2.4. Histological Analysis

For histological staining of paraffin-embedded tissue samples, slides after dewaxing, rehydration, antigen retrieval, and blocking were incubated with primary antibodies against NE (diluted 1 : 100, Santa Cruz, Cat number sc-55549) overnight at 4°C. After washing with PBS, slides were incubated with goat anti-mouse IgG H&L Alexa Fluor-488 (diluted 1 : 200, Abcam, Cat number ab150113). Nuclei were visualized with DAPI (0.4 *μ*g/mL in PBS, CST, Cat number 4083). The coverslips were mounted onto glass slides with Prolong Gold mounting agent (Molecular Probes, Cat number P36930). Images were captured using the Olympus Fluorescence Microscope (Upright BX61) under 10x lens (100-fold magnification).

### 2.5. Mouse Plasma Analysis

The plasma cell-free DNA was isolated by Serum/Plasma Circulating DNA Kit (TIANGEN, Cat number DP339), and DNA concentrations were determined using iQuant™ Broad Range dsDNA Quantification Kit (GeneCopoeia, Cat number N013); levels of plasma fibrinogen and NE were determined by ELISA method using mouse fibrinogen SimpleStep ELISA kit (Abcam, Cat number ab213478) and mouse neutrophil elastase ELISA kit (Bio-Swamp, Cat number MU30428), respectively.

### 2.6. Isolation of Neutrophils from Peripheral Human Blood

Peripheral blood from healthy donors was collected in K_2_·EDTA anticoagulant blood collection tubes and kept at room temperature before experiment. A signed informed consent was obtained from each donor, and the protocol was approved by Yangzhou Hospital of Chinese Medicine. The blood was used within two hours of drawing from the donors. Neutrophils were isolated using PolymorphPrep™ density gradient media (Axis-Shield, Cat number 1114683) according to the manufacturer's instructions with minor modifications. Red blood cells were then removed by ACK (ammonium-chloride-potassium) buffer (eBioscience, Cat number 00-4300-54). Isolated neutrophils were washed by HBSS buffer and resuspended in RPMI 1640 cell culture media without phenol red. Purity of cells was >95% as determined by Wright-Giemsa staining.

### 2.7. Measurement of Extracellular DNA Release by Plate Reader

To screen compounds that potentially inhibited NETosis, neutrophils were seeded at 3 × 10^4^ cells per well in a 96-well black, transparent bottom microplates (Costar, Cat number 3603) in phenol red-free RPMI media, and NETosis was monitored with changes in fluorescence intensity of a membrane-impermeable DNA-binding dye SYTOX™ Green (Invitrogen, Cat number S7020) indicative of DNA release. To quantify the activity of NETosis, NETotic index was used to indicate the extent of DNA release. Furthermore, we used Triton X-100 to disrupt the integrity of cell membrane, which caused a maximum exposure of nuclear and chromosome that could be completely accessible to DNA stain. Thus, the readout from the cells without incubation of DNA stain and incubated with DNA stain alone was considered as background and blank signals. Briefly, cells were preincubated with various compounds for 2 hours at 37°C, and DNase I (Thermo Scientific, Cat number 90083) was used as the NETosis inhibitor control. Subsequently, neutrophils were stimulated with 200 ng/mL of PMA (Sigma-Aldrich, Cat number P8139) for 4 hours to trigger maximal NET release in the presence of SYTOX Green (1 *μ*M). The readout from the cells incubated with nucleic acid stain and solvent DMSO was vehicle control. The fluorescence intensity was measured by EnSpire™ Multilabel Plate Reader (PerkinElmer, Ex/Em: 504/523 nm). To calculate NETotic index, fluorescence readout obtained from cells lysed with 0.5% (*v*/*v*) Triton X-100 was considered as 100% DNA release, and the index was calculated as the percentage of total values: NETotic index (%) = (RFU of vehicle control/experimental wells) × 100/RFU of Triton X-100 wells [26]. Drug combination effect was evaluated by CompuSyn (http://www.combosyn.com) based on the median-effect principle of Chou and Talalay. Combination index (CI) is a general expression and quantification of drug interaction, and CI values ranging from 0.1 to 0.3 signify strong synergism after combination therapy [27].

### 2.8. Microscopic Analysis of NETosis

NETosis was further confirmed by imaging extracellular DNA and the colocalization of neutrophil elastase, histone, and DNA. Neutrophils were seeded at 1 × 10^6^ cells per well on glass coverslips in 24-well plates and pretreated with various compounds and further activated with PMA then fixed with precooled acetone and rinsed three times with PBS. For DNA staining, cells were incubated with SYTOX Green alone (1 *μ*M) and coverslips were mounted onto glass slides with Prolong Gold mounting agent (Molecular Probes, Cat number P36930). Images were captured using the Olympus Fluorescence Microscope (Upright BX61) under 100x oil lens (1000-fold magnification) and analyzed using Volocity 6.0 software. For multiplex immunostaining assay, cells were permeabilized in 0.1% Triton X-100 and incubated with 1% BSA/PBS to block nonspecific antibody binding, followed by incubation with the antibody against Histone H1.0 (diluted 1 : 100, Abcam, Cat number ab125027) overnight at 4°C. After washing with PBS, cells were incubated with Goat anti-Rabbit IgG H&L Alexa Fluor-594 (diluted 1 : 1000, Cat number ab150080) for 2 hours at room temperature. The samples were further incubated with antibody against NE (diluted 1 : 100, Santa Cruz, Cat number sc-55549) overnight at 4°C. After washing with PBS, cells were incubated with goat anti-mouse IgG H&L Alexa Fluor-488 (diluted 1 : 1000, Cat number ab150113) for 2 hours at room temperature. Nuclei were visualized with DAPI (0.4 *μ*g/mL in PBS, CST, Cat number 4083). After mounting, cells were visualized using Olympus confocal laser scanning biological microscope (FluoView™ FV1000) under 60x oil immersion lens (600-fold magnification) and analyzed using FV10-ASW software.

### 2.9. Purified Enzymatic Activity Analysis

The inhibitory activity of indicated compounds on purified enzymes was performed by commercial kits according to instructions, including PAD4 inhibitor screening assay kit (Cayman Chemical, Cat number 700560), myeloperoxidase inhibitor screen assay kit (Cayman Chemical, Cat number 700170), and neutrophil elastase inhibitor screening kit (Sigma-Aldrich, Cat number MAK 213), which were all fluorescence-based methods for screening inhibitors of enzymatic activity. Fluorescence intensity was measured by EnSpire Multilabel Plate Reader (PerkinElmer) at Ex/Em: 575 nm/590 nm. After background subtraction, results were presented as 100% of activity or inhibition normalized to relative fluorescence units (RFU) of vehicle control.

### 2.10. NADPH Oxidase Activity Assay

The NADPH oxidase activity was performed with lucigenin- (bis-N-methylacridinium nitrate-) enhanced chemiluminescence method in the presence of its substrate NADPH. Lucigenin is a luminescence-generating reagent that interacts with NOX-derived superoxide anion, and the emitted luminescence can be quantitatively analyzed by a luminometer [28]. Specificity for non-NOX-mediated superoxide production was confirmed by adding a pan-NOX inhibitor diphenylene iodonium (DPI). Moreover, different from previous purified enzymatic assays, NADPH oxidase is quite complex with various subunits that normally require the whole cell machinery [29].

In short, aliquots of neutrophils or BGC-823 cells at a density of 3 × 10^4^ cells/mL cultured in 24-well plates were incubated with indicated compounds with or without PMA in the presence or absence of DPI (Sigma-Aldrich, Cat number D2926) for 1 hour. Subsequently, the cells were collected and washed once with ice-cold PBS. Cell pellets were then resuspended in 100 *μ*L of ice-cold Krebs-HEPES buffer with 20 *μ*M lucigenin (Sigma-Aldrich, Cat number M8010) containing 99 mM NaCl, 4.69 mM KCl, 2.5 mM CaCl_2_, 1.2 mM MgSO_4_, 25 mM NaHCO_3_, 1.03 mM KH_2_PO_4_, 5.6 mM D-(+)-glucose, and 20 mM Na-HEPES. 100 *μ*L of cell suspension was transferred to 96-well white plates with solid bottom (Costar, Cat number 3912). 20 *μ*L of NADPH (Sigma-Aldrich, Cat number N7505) balanced with Krebs-HEPES buffer was sequentially added to the well at final concentrations of 500 *μ*M. The emitted luminescence was detected by EnSpire Multilabel Plate Reader (PerkinElmer) every minute for 30 minutes, and the slope with 10 minutes for the trend line was defined as the relative NOX activity, expressed as luminescence unit (RLU)/minute.

### 2.11. Cell Viability and Apoptosis Assay

Isolated neutrophils (5 × 10^6^ cells/mL) were cultured in 6-well plates in RPMI 1640 supplemented with 10% FBS and treated with Sal B, DHT I, Sal B combined with DHT I, and DSI for 0, 12, and 24 hours, respectively. At stated time points, neutrophils were resuspended in 500 *μ*L of binding buffer and labeled with FITC-Annexin V-propidium iodide (PI) before analysis on BD LSRFortessa™ cytofluorometer (BD Biosciences).

For tumor cells, the cell viability was measured by MTT assay. BGC-823 cells (1 × 10^4^ cells/well) were seeded in 96-well plate and treated with various concentrations of indicated treatment for 48 hours and subsequently added by 20 *μ*L/well of 0.5 mg/mL MTT (Sigma-Aldrich, Cat number M2128). After another incubation of 4 hours, the medium was carefully aspirated and replaced with 150 *μ*L/well of DMSO. After brief shaking for 15 minutes, the absorbance was measured with EnSpire Multilabel Plate Reader (PerkinElmer) at 480 nm.

### 2.12. Western Blot Analysis

For experiments requiring making of cell lysates for analysis, aliquots of neutrophils in 1.5 mL Eppendorf tubes at a density of 5 × 10^6^ cells/mL were incubated with indicated compounds for 2 hours and further stimulated with PMA for 4 hours on an oscillator in carbon dioxide incubators. Subsequently, the tubes containing the cells were centrifuged and washed once with ice-cold PBS and lysed with RIPA buffer (150 mM NaCl, 50 mM Tris-HCl, pH 8.0, 0.5% sodium deoxycholate, 0.1% SDS, 1% NP-40, 2 mM EDTA pH 8.0, and 0.2% sodium fluoride) containing protease and phosphatase inhibitors. Equal amounts of cell lysates (25 *μ*g) were resolved by 10% SDS-PAGE and transferred onto PVDF membranes. Membranes were incubated with monoclonal antibodies against histone H3 (citrulline R2 + R8 + R17) at dilution of 1 : 1000 and GAPDH (Abcam, Cat number ab8245) at dilution of 1 : 10000. Membranes were incubated with HRP-conjugated anti-rabbit or mouse IgGs (Proteintech, Cat number SA00001-2 and SA00001-1) at dilution of 1 : 10000 followed by enhanced chemiluminescence (Millipore, Cat number WBKLS0500) and visualized with a ChemiDoc XRS system (Bio-Rad).

### 2.13. Statistical Analysis

Data are shown as averages with standard deviations and evaluated using either two-tailed Student's *t-*test or one-way ANOVA followed by post hoc Dunnett's test. Differences where *p* < 0.05 were considered statistically significant.

## 3. Results

### 3.1. Danshen Attenuates Experimental Pulmonary Metastasis

**Figure 1:**
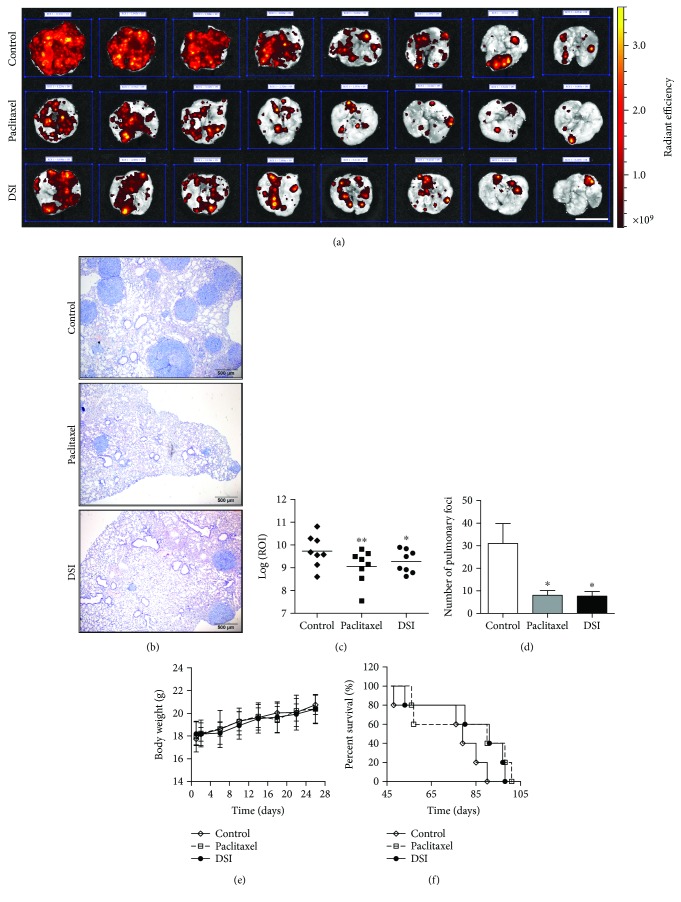
Danshen inhibits lung metastases *in vivo*. (a) BGC-823 cells transduced with RFP protein were injected into the tail vein of BALB/c athymic nude mice, and the ex vivo fluorescence signal of the surface lung metastases was quantified after four weeks. The ex vivo images of isolated lung from vehicle (normal saline, control, i.p.), paclitaxel (10 mg/kg, every 3 days for a total of 9 injections), or danshen injection- (DSI-, 780 mg/kg, q.d. for a total 28 injections) treated mice (*n* = 8 per group) were shown in (a), and representative lung H&E sections of metastases (×100) were shown in (b). Scale bar indicates 1 cm (a) and 500 *μ*m (b), respectively. (c) Quantification (mean ± SD, *n* = 8) of logarithmic transform of ROI (log 10) in the control and treated groups are portrayed. Statistical comparison between control and treated groups was carried out using Student's *t*-test (^∗^ *p* = 0.004 between control and paclitaxel and ^∗∗^ *p* = 0.038 between control and DSI). (d) Quantification of foci number (mean ± SEM, *n* = 8) using histological analysis of metastatic tumors in lung slides. Statistical comparison between control and treated groups was carried out using Student's *t*-test (^∗^ *p* = 0.024 between control and paclitaxel and ^∗∗^ *p* = 0.022 between control and DSI). (e) Changes of body weight after drug treatment were recorded every day. (f) Survival analysis of mice with different treatments from the first four weeks and then free of drug intervention until death. The data were analyzed by the Kaplan-Meier survival analysis with the log-rank test (*p* = 0.229 between control and paclitaxel and *p* = 0.094 between control and DSI, *n* = 5).

A commonly used hematogenous spread model is inoculation of tumor cells via the tail vein to implant cells in a mouse lung. Thus, we employed this murine model to investigate the antimetastatic effects of Danshen, and we administrated mice with DSI (780 mg/kg, q.d. for a total 28 injections) as its injection form with much higher bioavailability. Animals received paclitaxel (10 mg/kg, every 3 days for a total of 9 injections) as a positive treatment control. To visualize and quantify the metastatic foci, we applied RFP-transduced BGC-823 cells that could be visualized by the live-imaging system. We sacrificed animals (*n* = 8 per group) after four weeks to check the metastatic foci. In another experiment, all the treatments were stopped after four weeks for recording survival (*n* = 5 per group). As shown in Figure 1(a), we observed reduced fluorescent signal in mouse lung after DSI and paclitaxel treatment, which was further confirmed by histological inspection (Figure 1(b)). In addition, paclitaxel and DSI could significantly attenuate log 10-transformed fluorescent intensity of ROI (Figure 1(c)) and number of metastatic foci (Figure 1(d)) without affecting body weight (Figure 1(e)). Though the log-rank test of survival analysis was not statistically significant, we observed that all animals died within 3.5 months, among which those animals that received paclitaxel and DSI potentially prolonged survival with 90 and 91 days of median survival time compared with 79 days of that from the control group (Figure 1(f)). In sum, Danshen can limit the metastatic progression in a mouse model of experimental pulmonary metastasis of tumor-bearing mice.

### 3.2. Danshen Inhibits Neutrophil Recruitment in Tumor-Bearing Mice

**Figure 2:**
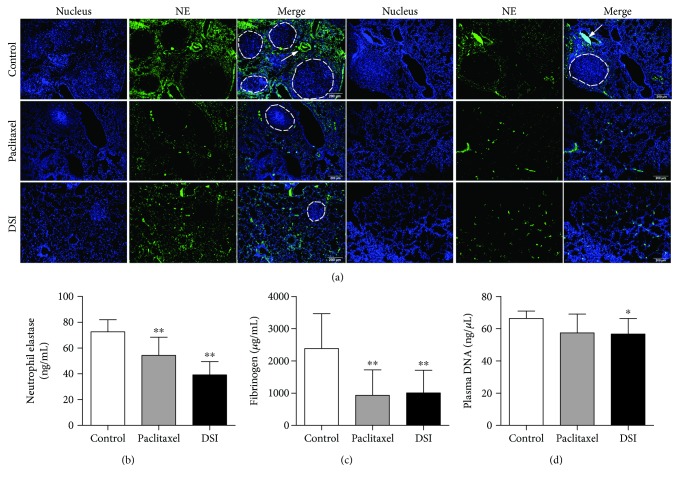
Danshen antagonizes neutrophil functions in tumor-bearing mice. (a) Representative microscopic images of two independent paraffin-embedded lungs from samples from two tumor-bearing mice under 100-fold magnification. Fluorescence staining of sections with NE (green) combined with DAPI (blue). Scale bar indicates 200 *μ*m. The plasma levels of NE (b) and fibrinogen (c) were determined by ELISA assay. Data were expressed as mean ± SD. Statistical comparison between control and treated groups was carried out using Student's *t*-test (^∗∗^ *p* < 0.01 between control and paclitaxel and ^∗∗^ *p* < 0.01 between control and DSI). The cell-free DNA (d) was determined based on the fluorescence method (^∗^ *p* = 0.026 between control and DSI).

Importantly, neutrophils have a fundamental role in inflammatory responses and their contribution to tumorigenesis is still controversial. In the experimental pulmonary metastasis mouse model, we examined recruitment of neutrophils to metastatic sites using immunofluorescence analysis of NE as a marker of neutrophil activity. Our data suggested that there were potentially increased neutrophil accumulation in the microvessels and distribution surrounding the tumor nodules (indicated by the white arrows) whereas lung sections from paclitaxel and DSI treatment showed less NE deposition around metastatic sites (Figure 2(a) and Supplementary Figure 1). Besides, DSI significantly downregulated the plasma levels of NE (Figure 2(b)), fibrinogen (Figure 2(c)), and cell-free DNA (Figure 2(d)), suggesting reduced proinflammatory state and risk of thrombosis in tumor-bearing mice. Taken together, our data further demonstrate that neutrophils are involved in the antimetastatic effect of Danshen.

### 3.3. Danshen-Derived Compounds Prevent PMA-Induced NETosis

**Figure 3:**
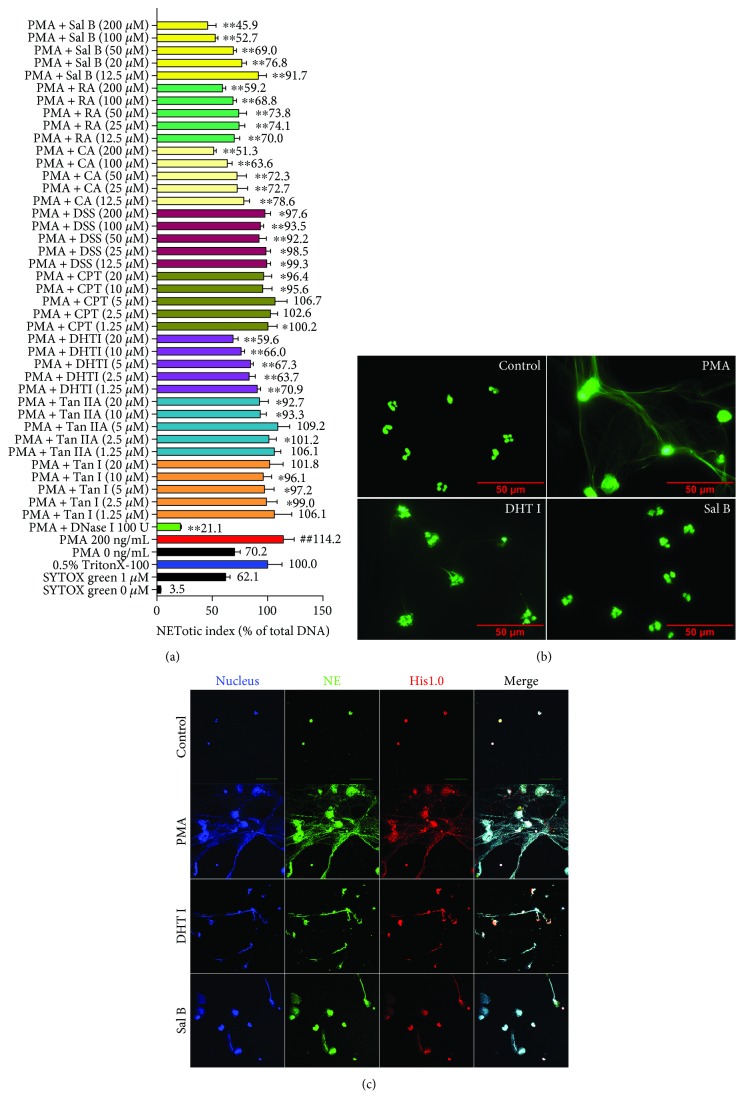
Active components derived from Danshen impair NET formation *in vitro*. (a) Screening natural inhibitors from eight primary components isolated from Danshen to PMA-induced NETosis. NETotic index was used to indicate the extent of DNA release during NETosis. Extracellular DNA release from human neutrophils was quantified by a membrane impermeable DNA-binding dye SYTOX Green. Fluorescence readout obtained from cells lysed with Triton X-100 was considered as 100% DNA release. DNA dissolving drug DNase I served as positive treatment control (^##^ *p* < 0.01 between 0 ng/mL of PMA treatment and 200 ng/mL of PMA treatment, while ^∗^ *p* < 0.05 or ^∗∗^ *p* < 0.01 between 200 ng/mL of PMA treatment and drug treatment). (b) Neutrophils were pretreated with or without Sal B (200 *μ*M) or DHT I (20 *μ*M) for 2 hours and further incubated with 200 ng/mL of PMA for 4 hours; the structures of NETs were observed by fluorescent microscopy for SYTOX Green staining in formalin-fixed neutrophils at 1000x magnification (scale bar represents 50 *μ*M). (c) NETs were further confirmed by a multicolor fluorescence immunostaining of DNA-histone-MPO complexes at 600x magnification (scale bar represents 20 *μ*M).

To investigate whether the compounds derived from Danshen are able to interfere with the process of NET formation, we used PMA, an activator of PKC to stimulate NET formation and SYTOX Green nucleic acid stain for monitoring DNA release. DNase I served as positive treatment control. We obtained eight well-known biological active compounds from Danshen (CA, DSS, RA, Sal B from the hydrophilic fraction and Tan I, DHT I, Tan IIA, and CPT from the lipophilic fraction, resp.) to screen their potential inhibitory activities on NET formation. As the natural content of the hydrophilic fraction is constitutively more abundant than that of the lipophilic fraction in Danshen [23], we differentially set 10-fold variation of dose range between the two fractions (12.5, 25, 50, 100, and 200 *μ*M for the hydrophilic fraction and 1.25, 2.5, 5, 10, and 20 *μ*M for the lipophilic fraction) *in vitro*. As a result, the hydrophilic fraction prevalently exhibited higher inhibitory activity with concentration-dependent response than the lipophilic fraction on PMA-induced NETosis. Additionally, Sal B and DHT I demonstrated their strong abilities to inhibit NETosis among the two fractions in Danshen (Figure 3(a)).

Subsequently, we confirmed the histological alterations during NETosis. Under 200 ng/mL of PMA stimulus, neutrophils were characterized with remarkable swollen nuclei and presence of NET-like structures of extracellular DNA whereas Sal B (200 *μ*M) and DHT I (20 *μ*M) could greatly reverse PMA-triggered DNA release and attenuated delobulation of neutrophil nuclei (Figure 3(b)). Furthermore, the levels of extracellular chromatin fiber-binding proteins activated during NETotic response were visualized by confocal imaging of colocalization of MPO and His1.0 with DNA, which were also sensitive to Sal B (200 *μ*M) and DHT I (20 *μ*M) treatment. Together, Danshen is a pooled library of potential inhibitors of PMA-triggered NET formation.

### 3.4. Activity of Danshen-Derived Compounds Is Required for NOX-Dependent NETosis

**Figure 4:**
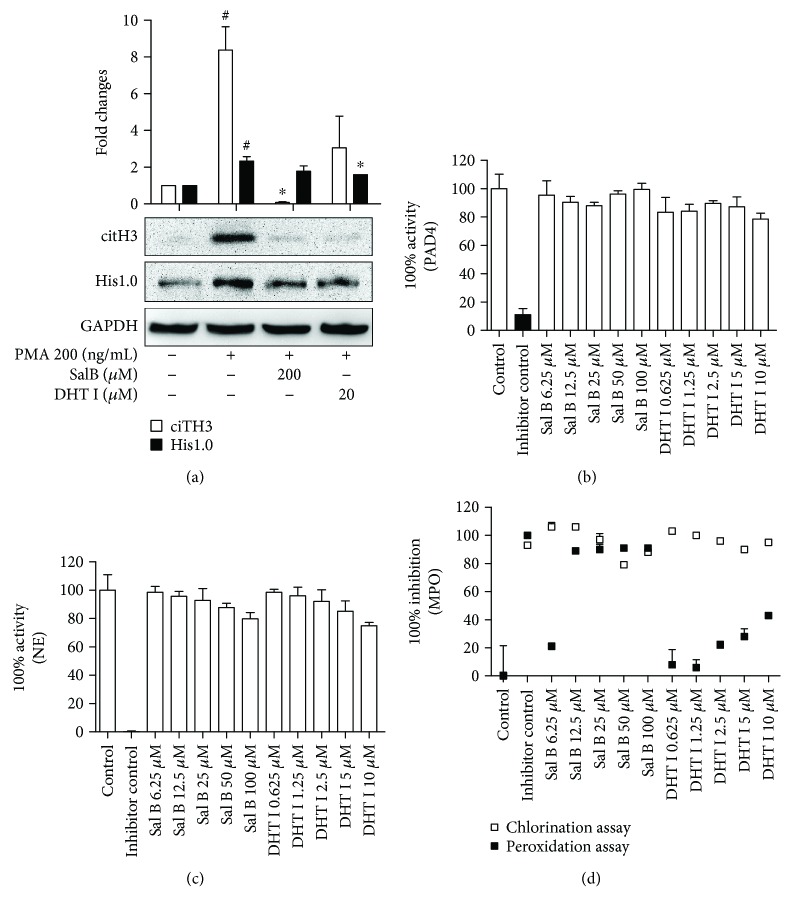
Sal B and DHT I selectively inhibit specific NET-associated enzymes. (a) The effects of Sal B and DHT I on citH3 by Western blot analysis. Protein levels were quantified with NIH ImageJ software and were normalized with GAPDH. Graphs show mean ± SD from two independent blots. ^#^ *p* < 0.05 between control and PMA, while ^∗^ *p* < 0.05 between PMA and PMA plus drug treatment. The effects of Sal B and DHT I on recombinant PAD4 activity (b) or NE (c) or MPO (d) using fluorescence-based methods provided by commercial kits. The potential effects were expressed either 100% inhibition or 100% activity normalized to vehicle controls in triplicate.

We next sought to identify the specific enzymes responsible for mediating the inhibitory effects of Sal B and DHT I on NETosis. Initially, we further assessed the levels of NETotic marker citH3 upon Sal B and DHT I treatment. In consistent with previous results, hypercitrullination of histone H3 was observed in PMA-incubated neutrophils whereas Sal B and DHT I could prevent the process as expected (Figure 4(a)). By screening of a number of key enzymes including PAD4, NE, MPO, and NOX that have been shown to be activated in PMA-induced NETosis, we found that Sal B and DHT I failed to target for purified PAD4 (Figure 4(b)) and NE (Figure 4(c)), but strongly inhibited MPO and NOX activities. More specifically, Sal B (6.25–100 *μ*M) showed a more than 50% inhibitory effect on both chlorination and peroxidation activities of purified MPO, while the maximum dose DHT I (10 *μ*M) approximately showed 50% inhibitory activities on peroxidation activity in a dose-dependent manner (Figure 4(d)).

**Figure 5:**
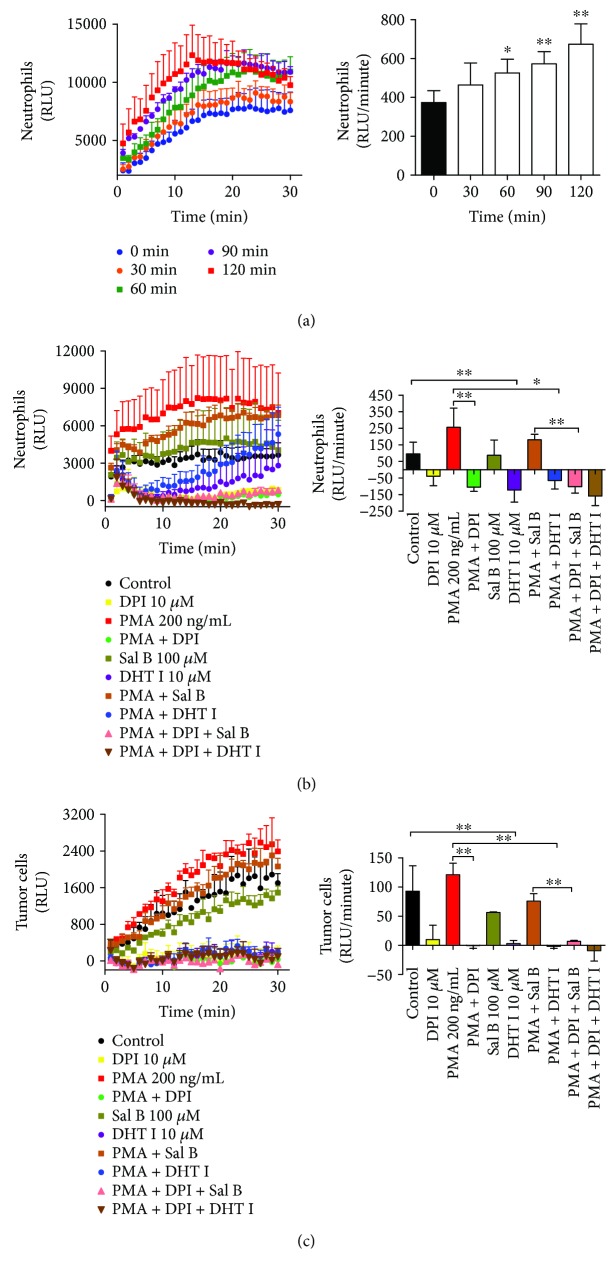
Sal B and DHT I inhibit NOX activities in neutrophils and tumor cells. (a) Human neutrophils were incubated with 200 ng/mL of PMA with various time courses (0, 30, 60, 90, and 120 minutes), and NOX activity was detected using lucigenin chemiluminescence assay. The NOX activity was expressed as luminescence unit (RLU)/minute in 10 minutes (^∗^ *p* < 0.05 or ^∗∗^ *p* < 0.01 between 0 minute and other time courses). The effect of Sal B and DHT I on NOX activity in neutrophils (b) or BGC-823 gastric cancer cells (c) in the presence or absence of NOX inhibitor DPI was measured by lucigenin chemiluminescence assay. ^∗^ *p* < 0.05 or ^∗∗^ *p* < 0.01 as shown in the indicated comparison.

NOX2 is dominantly expressed in neutrophils, and PMA is known to stimulate NOX-dependent NETotic response [30]. NOX activity was determined in intact whole cell machinery measured using lucigenin-enhanced chemiluminescence. As illustrated in Figure 5(a), PMA (200 ng/mL) elevated NOX activity in a time-dependent manner within 1 hour incubation time. Thus, we cotreated neutrophils with PMA in the presence or absence of Sal B (100 *μ*M) or DHT I (10 *μ*M) for 1 hour to examine the effect of Sal B and DHT I on NOX activity. Our data showed that NOX inhibitor DPI (10 *μ*M) substantially deactivated both quiescent and PMA-induced NOX activities. In comparison, Sal B (100 *μ*M) hardly whereas DHT I (10 *μ*M) strongly suppressed NOX activity regardless of the presence or absence of PMA. Moreover, DPI further enhanced the effect of Sal B and DHT I (Figure 5(b)). As tumor cells also undergo aberrant control of NOX expression [31], we next asked whether Sal B and DHT I could disrupt NOX activity in tumor cells. As we found, the chemiluminescence signal triggered by PMA in BGC-823 gastric cancer cells was much lower than that in neutrophils, suggesting distinct tissue-derived NOX kinetics. In addition, DHT I but not Sal B was still able to negatively modulate NOX activity in tumor cells (Figure 5(c)). Therefore, these results highlight that the activity of compounds from Danshen primarily targets oxidative burst-mediated NET formation.

### 3.5. Danshen-Derived Compounds Show Fundamentally Different Interactive Manners in Neutrophils and Tumor Cells

**Figure 6:**
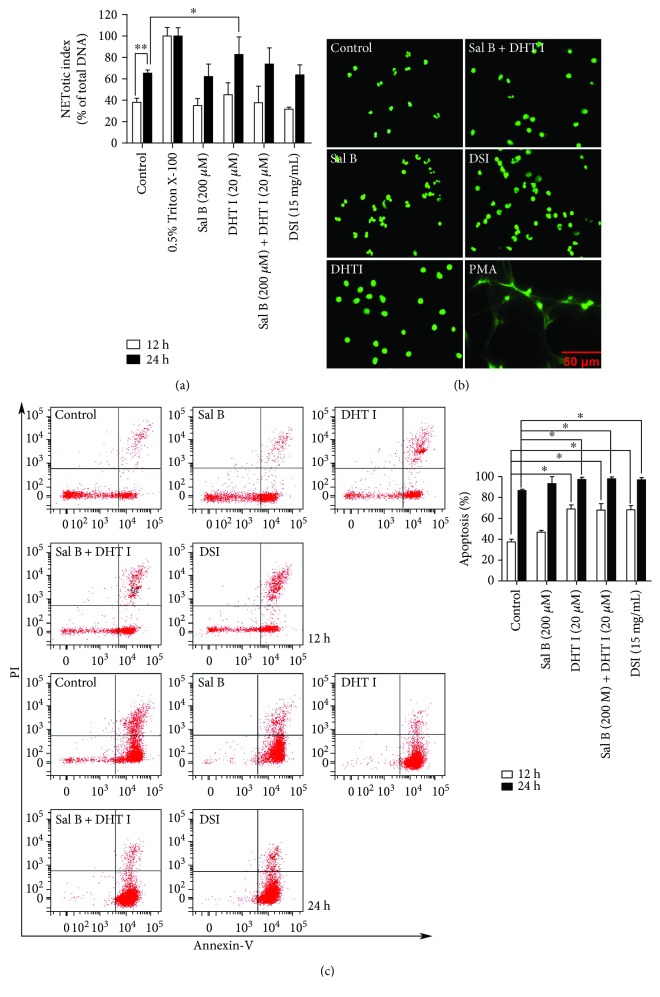
Danshen and its components promote the spontaneous apoptosis of neutrophils *in vitro*. (a) The effects of Sal B, DHT I, Sal B plus DHT I, or DSI on the NETotic activity of neutrophils for 12- and 24-hour treatments by plate reader assay. ^∗^ *p* < 0.05 or ^∗∗^ *p* < 0.01 as shown in the indicated comparison. (b) Nuclear morphology after 24-hour drug treatment was assessed by cell morphology at 400x magnification, scale bar represents 50 *μ*M. (c) The effects of Sal B, DHT I, Sal B plus DHT I, or DSI on neutrophil apoptosis for 12- and 24-hour treatments by flow cytometry after Annexin V/PI labeling. Samples were run in duplicate, and 10,000 events were captured within the neutrophil gate each time. The percentage of apoptotic cells (%) was cells labeled with Annexin V (+)/PI (−) and Annexin V (+)/PI (+). ^∗^ *p* < 0.05 as shown in the indicated comparison.

To systematically evaluate the integrative effect of Danshen-derived compounds in inflammatory and malignant conditions, we thus treated neutrophils and BGC-823 gastric cancer cells in combination with Sal B and DHT I at a constant 10-fold of combination dose ratio. Since neutrophils have a short lifespan and undergo spontaneous apoptosis within hours *in vitro* [32, 33], even a slight effect on the apoptosis can have a major impact on the viability on neutrophils. Moreover, chronic toxicity studies have showed that long-term injection of DSI or Danshen applied as daily food has no effect on the counts of neutrophils [34–36], leaving the effects of Danshen on cell viability uncertain and in need of further research. Neutrophils can die by various forms of cell death, and we need to confirm that whether Danshen and its components could modulate neutrophil viability either by NETosis or by apoptosis. Notably, the cell-impermeable nucleic acid stain is used to track DNA release during NETosis, which can be highly permeant to apoptotic cells. Therefore, it is important to discriminate the readout of NETotic assay that is truly derived from NETotic cells or apoptotic cells. To this end, we further clarified the morphology changes of neutrophils during NETosis (nuclear decondensation concurrent with extracellular DAN release) or apoptosis (nuclear condensation without extracellular DAN release). As a result, we found that the highlighted component DHT I (20 *μ*M) had a slight effect (*p* < 0.05) on activating NETotic activity in a time-dependent manner (Figure 6(a)). However, we failed to detect typical extracellular DNA release after 24-hour treatment of DHT I by morphology study (Figure 6(b)). Thus, we further confirmed neutrophil apoptosis using flow cytometry by Annexin V and PI staining. Our data showed that normal neutrophils underwent time-dependent spontaneous apoptosis within 12–24 hours (Figure 6(c)), while DHT I, Sal B plus DSI, and DSI displayed significant neutrophil apoptosis-inducing effect (*p* < 0.05). This is a reflection of one of the other essential functions of Danshen by inducing the biology of neutrophil apoptosis.

**Figure 7:**
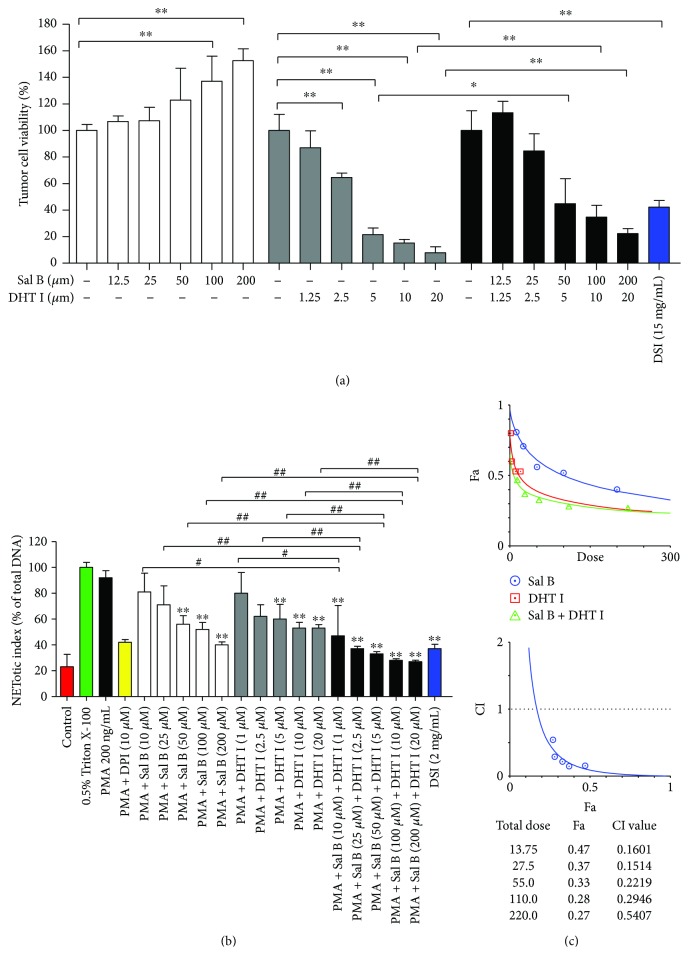
Sal B and DHT I synergistically attenuate the formation of NETs. (a) The effects of DSI, Sal B, DHT I, or the combination of Sal B and DHT I on the cell vitality in BGC-823 gastric cancer cells for 48-hour treatment. ^∗^ *p* < 0.05 or ^∗∗^ *p* < 0.01 as shown in the indicated comparison. (b) Human neutrophils were activated by PMA (200 ng/mL) in the presence of DPI, DSI, Sal B, DHT I, or combination of Sal B or DHT I on NETosis by plate reader assay. ^∗∗^ *p* < 0.01 between control and drug treatment. ^#^ *p* < 0.05 or ^##^ *p* < 0.01 as shown in the indicated comparison. (c) Analysis of drug combinations using median-effect equation and CompuSyn software. ^∗^ *p* < 0.05 or ^∗∗^ *p* < 0.01 as shown in the indicated comparison.

Next, we investigated the effect of Danshen on the cell viability of tumor cells. Interestingly, DHT I had prominent cytotoxicity effect on tumor cells after 48-hour treatment while Sal B partially exerted cytoprotective role in DHT I-induced cell death. DSI (15 mg/mL, equivalent to 0.0048 mg/mL of Sal B in DSI) also inhibited tumor cell growth *in vitro* (Figure 7(a)). In another round of plate reader assay, DSI (15 mg/mL) had a less than 50% inhibitory effect on NET formation, while Sal B and DHT I collectively inhibited NET formation and showed strong synergistic drug interaction with the combination index less than 0.3 (Figures 7(b) and 7(c)). The ED_50_ was strikingly reduced to 6.9 *μ*M as potent as DPI. Therefore, Danshen-derived compounds play complex interactions in multiple cell types, and these actions can be either synergistic or antagonistic.

## 4. Discussion

Two major fractions of active components featured by phenolics and diterpenoids from Danshen are completely different in chemical structures. Previous studies can be selectively focused on the activity of either phenolic acids or tanshinones or single compounds. In the present work, we revealed very interesting findings on the mechanism of actions of both phenolic acids and tanshinones on biological system. Although neutrophils have a significant impact on the tumor microenvironment, the potential roles of NETotic events in the course of tumor metastasis have recently become a focus of attention. We observed accumulation of neutrophils in the adjacent vascular vessels which might play critical roles during the colonization of tumor in the experimental lung metastasis mice model. Studies have shown that Danshen ingredients can attenuate neutrophil migration, infiltration, and adhesion with endothelial cells in inflammatory responses in a number of models of inflammation [37, 38]. Importantly, we advanced current knowledge of Danshen on tumor metastasis in association with neutrophils by other mechanisms on NET formation.

Although the molecular pathways leading to NET formation remain to be fully characterized, the process of NETosis is known to involve activation of PKC, which then phosphorylates the p40^phox^ and p47^phox^, leading to assembly of the NOX complex [39]. Some tumor cell-derived factors such as colony-stimulating factor (G-CSF) that induces NET formation have been recognized [40]. IL-8 and LPS can drive NETosis, but their ability to reliably reproduce NETosis is questionable [41]. Using PMA as a potent NET inducer, we screened phenolic acids and tanshinones responsible for NET inhibitors. We noticed that phenolics exhibited a more remarkable activity to reduce the extent of DNA release. It was reasonable that the effective doses of phenolics were 10-fold higher than those of tanshinones to attain equal levels of inhibitory activity of NETs upon dose ratio of the two fractions according to the natural proportions in Danshen roots (75 mg/g for phenolics and 8 mg/g for tanshinones, resp.) [42]. Notably, the content of tanshinones varies a lot in diverse pharmaceutical products of Danshen and is almost undetectable in DSI [43]. Thus, the antitumor role of DSI in the present study was primarily mediated by phenolic constituents.

Overall, we have excluded PAD4 and NE as direct drug targets of Sal B and DHT I, while the two components both exhibited different properties on key enzymes involved in oxidative burst during NETosis. We found that Sal B and DTH I predominantly disrupted MPO and NOX activity, respectively. Thus, Danshen may preferentially interfere with NETs at the earlier stage of NET formation. MPO and NOX are the most abundant proteins and major source of ROS in neutrophils that mediate the PMA-induced formation of NETs [44]. Phenolic compounds are a large group of antioxidants in herbal medicine with potent free radical scavenging abilities [24]. Contrarily, tanshinones have been reported to increase the intracellular production of ROS in macrophages [45]. It was intriguing how multiple ingredients from Danshen modulate the ROS-related effects on various cell type and pathological conditions.

To discriminate that the inhibitor specifically interferes with the chlorination and/or peroxidation cycle of MPO or the inhibitor simply acts as a scavenger for HOCl, it is important that when screening for MPO inhibitors, both of the chlorination and peroxidation activities should be tested. The peroxidase cycle does not produce HOCl, and putative MPO inhibitors might be more selective in inhibiting MPO activity towards peroxidase substrates [46]. Therefore, our current data on Sal B and DHT might be specific as potential MPO inhibitors primed for peroxidase substrates. NOX activity is controlled by a complex regulatory system and difficult to test in a purified enzyme system. NOX catalyzes the conversion of cytoplasmic NADPH to NADP^+^ with concomitant transfer of electrons through the flavin adenine dinucleotide (FAD) domain and iron-heme prosthetic groups to oxygen molecules, resulting in formation of superoxide anions, which can be detected by chemiluminescent probe lucigenin. Furthermore, lucigenin specifically reacts with superoxide, but not MPO-derived ROS [47], which can ensure species-specific oxidizing enzymes in drug screening. To eliminate the potential effects of Sal B and DHT I on cell viability, we measured a shorter time course that could significantly activated NOX activity (only 1 hour). From current data, Sal B and DHT I displayed an inhibitory effect on NOX which could be further intensified by DPI in neutrophils. Furthermore, NOX was less active in tumor cells than in neutrophils, because PMA hardly activated NOX activity in tumor cells. Nevertheless, we obtained consistent results with Sal B and DHT I, implying that cell type-independent inhibitory effects on NOX activity are mediated by Sal B and DHT I.

Since Sal B and DHT I preferentially inhibited MPO and NOX enzymatic activity, respectively, we speculated that the two components could be coupled with each other to antagonize NET formation to maximum potential. In agreement with our hypothesis, Sal B and DHT I collectively resulted in a more significant inhibition of NETosis under subED_50_ doses compared to Sal B or DHT I treatment alone. However, Sal B or DHT I had opposite roles in regulating cell survival of neutrophils and tumor cells and the cytotoxic effect of tanshinones has been defined by production of ROS essential for killing tumor cells [45], but less toxic to normal cells and protect normal cells from oxidative injury [48, 49]. In general, elevated ROS levels and adaptation to ROS stress in cancer cells support tumor survival while overproduction of ROS can be suicidal to normal cells including neutrophils. As both fractions of Danshen show antioxidative burden in NETosis, our preliminary data provide valuable insights that aqueous fraction of Danshen represented by Sal B might be prepared to protect tanshinones from presumable ROS insults without harmful effects on normal cells. Moreover, we found that DHT I but not Sal B exhibited proapoptotic effect on neutrophils. Recent advances have shed light on exploiting the biology of neutrophil apoptosis to drive resolution of inflammation and minimize tissue damage [50, 51], and compelling evidence revealed that the active components tanshinones had no effect on total neutrophil numbers but increased the neutrophil apoptosis at the site of tissue injury [52]. It is now reasonably well understood that the best-described mechanism by which neutrophils are removed from inflammatory tissues is apoptosis. Delayed neutrophil apoptosis enhances NET formation [53], and we ask whether promotion of neutrophil apoptosis by DHT I is one of the mechanisms that rescue neutrophils from NETosis. Thus, diversion of neutrophils from NETosis to apoptosis by tanshinones might have some therapeutic benefit to prevent excessive release of neutrophil contents during NETosis. Danshen could modulate the unique redox and survival regulatory mechanisms that might be an effective strategy to treat different disease systems in both cancer and inflammatory diseases.

**Figure 8:**
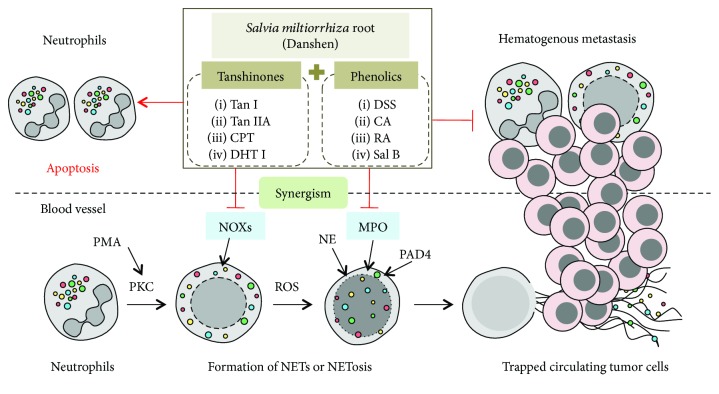
Summary of the main findings.

In conclusion, Danshen manifests a therapeutic role as an antitumor agent by preventing early phase of NETs (Figure 8). Our data, at least partially, propose the systematic therapeutic management of Danshen as an integrative medication that could bring a better outcome. Each individual component can select their own pathway to evolve a special integration through a great deal of crosstalk at multiple pharmacological levels, and the mode of action (MOA) of CMM is far more complicated than our current understanding well defined by a multicomponent, multitarget medication.

## Data Availability

The datasets generated during and/or analyzed during the current study are available from the corresponding author on reasonable request.

## Supplementary Materials

Supplementary 1 Supplementary Table 1: chemical information.

Supplementary 2 Supplementary Document 1: photocopy and English translation of the certificate of analysis (COA) of DSI.

Supplementary 3 Supplementary Figure 1: supplementary immunostaining images for NE expression associated with metastases.
