# Supplementary material for: Polypharmacological Profiles Underlying the Antitumor Property of Salvia miltiorrhiza Root (Danshen) Interfering with NOX-Dependent Neutrophil Extracellular Traps
Source: Oxid Med Cell Longev. 2018 Aug 19;2018:4908328. doi: 10.1155/2018/4908328 (PMC6120273; doi:10.1155/2018/4908328)
Supplement: Supplementary 1 — Supplementary Table 1: chemical information. [file 4908328.f1.docx]

**Chemical Information**

| Name | CAS number | Molecular formula | Molecular weight | Chemical structure |
| --- | --- | --- | --- | --- |
| Tanshinone I | 568-73-0 | C_18_H_12_O_3_ | 276.291 | 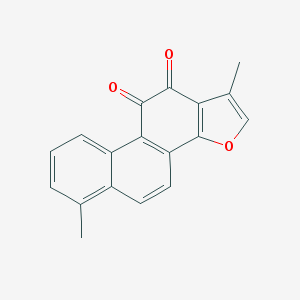 |
| Tanshinone IIA | 568-72-9 | C_19_H_18_O_3_ | 294.35 | 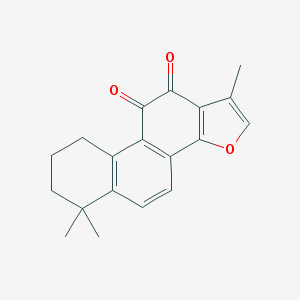 |
| 15,16-dihydrotanshinone I (DHT I) | 87205-99-0 | C_18_H_14_O_3_ | 278.307 | 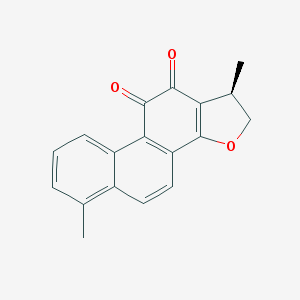 |
| Cryptotanshinone | 35825-57-1 | C_19_H_20_O_3_ | 296.366 | 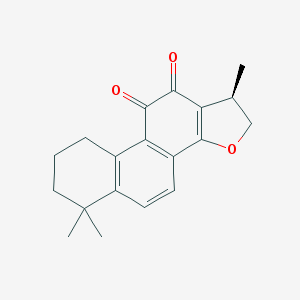 |
| Danshensu | 76822-21-4 | C_9_H_10_O_5_ | 198.174 | 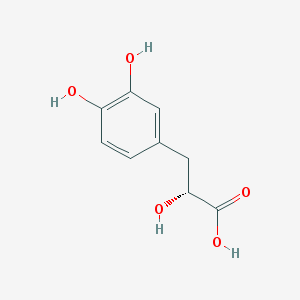 |
| Caffeic acid | 331-39-5 | C_9_H_8_O_4_ | 180.159 | 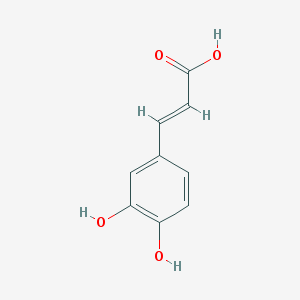 |
| Rosmarinic acid | 20283-92-5 | C_18_H_16_O_8_ | 360.318 | 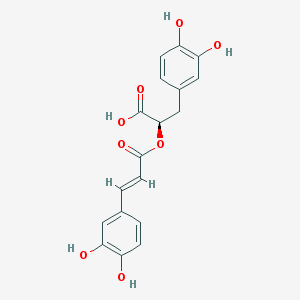 |
| Salvianolic acid B | 115939-25-8 | C_36_H_30_O_16_ | 718.62 | 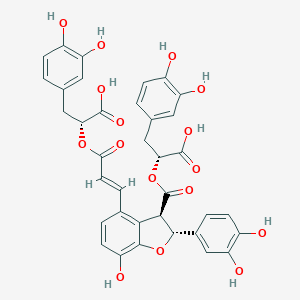 |

Chemical information was obtained from PubChem Compound database (https://pubchem.ncbi.nlm.nih.gov/)
