# Supplementary material for: Polypharmacological Profiles Underlying the Antitumor Property of Salvia miltiorrhiza Root (Danshen) Interfering with NOX-Dependent Neutrophil Extracellular Traps
Source: Oxid Med Cell Longev. 2018 Aug 19;2018:4908328. doi: 10.1155/2018/4908328 (PMC6120273; doi:10.1155/2018/4908328)
Supplement: Supplementary 2 — Supplementary Document 1: photocopy and English translation of the certificate of analysis (COA) of DSI. [file 4908328.f2.docx]

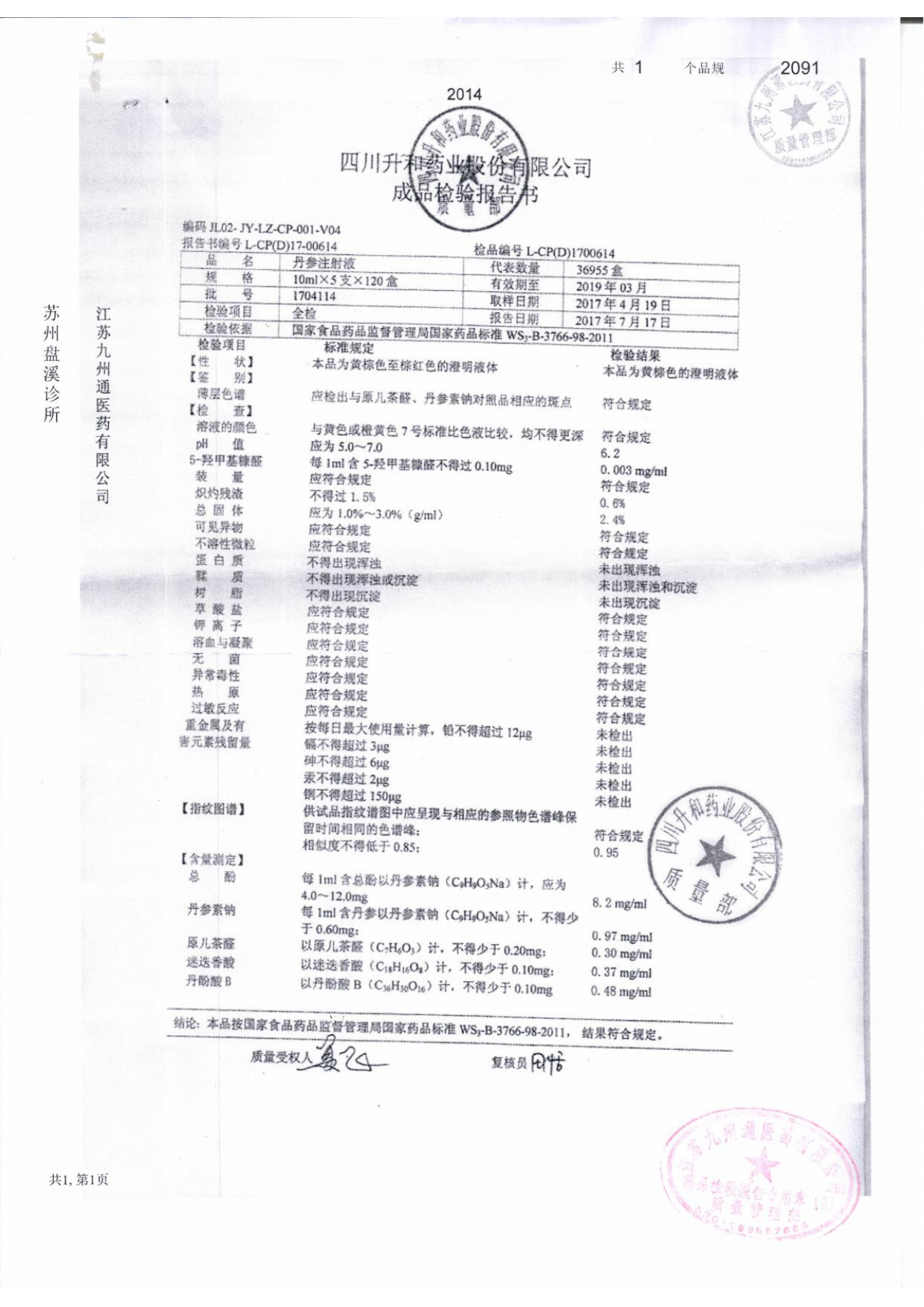


**Certificate of Analysis**

**Manufacture:** Sunnyhope Pharmaceutical Co., Ltd (Sichuan, China)

**Document ID:** JY-LZ-CP-001-V04

**Certificate ID:** L-CP(D)17-00614

**Test samples ID:** L-CP(D)1700614

| **Product Name** | Danshen Injection | **Sample size** | 36955 boxes |
| --- | --- | --- | --- |
| **Bulk Package Size** | 10 mL × 5 bottles ×120 boxes | **Expiration date** | March, 2019 |
| **Lot number** | 1704114 | **Date of sampling** | April 19,2017 |
| **Analytic item** | All the required items | **Date of data reporting** | July 17, 2017 |
| **Quality standard** | National drug standards (No. WS3-B-3766-98-2011) of China Food and Drug Administration (CFDA) | | |

**Analytical Data**

| Analytic item | | | Quality standard | Results |
| --- | --- | --- | --- | --- |
| **1. Appearance** | | | | |
| Clear liquid with brownish-yellow or reddish-brown color | | | | clear liquid with brownish-yellow color |
| **2. Identification** | | | | |
| 2.1 TLC | | danshensu sodium salt and protocatechuic aldehyde spots will appear at the corresponding position of reference standards | | qualified |
| 2.2 Appearance of solution | | no deeper than EP color standard/brownish-yellow reference solution BY7 | | qualified |
| 2.3 PH | | 5.0-7.0 | | 6.2 |
| 2.4 5-hydroxymethylfurfural | | less than 0.10 mg/mL | | 0.03 mg/mL |
| 2.5 Volume variation | | meets the requirements | | qualified |
| 2.6 Residue on ignition | | less than 1.5% | | 0.6% |
| 2.7 Total solid materials | | 1.0%-3.0% (g/mL) | | 2.4% |
| 2.8 Visible particles | | meets the requirements | | qualified |
| 2.9 Insoluble particle | | meets the requirements | | qualified |
| 2.10 Proteins | | without cloudy appearance | | qualified |
| 2.11 Tannin materials | | without cloudy appearance or precipitates | | qualified |
| 2.12 Resin materials | | no precipitates | | qualified |
| 2.13 Oxalate salts | | meets the requirements | | qualified |
| 2.14 Potassium salts | | meets the requirements | | qualified |
| 2.15 Hemolysis and platelet aggregation/coagulation | | meets the requirements | | qualified |
| 2.16 Sterile | | meets the requirements | | qualified |
| 2.17 Abnormal toxicity | | Abnormal toxicity | | qualified |
| 2.18 Bacterial endotoxin | | meets the requirements | | qualified |
| 2.19 Allergic reaction | | meets the requirements | | qualified |
| 2.20 Heavy metals and residual metal elements | | lead: less than 12 μg;  cadmium: less than 3 μg;  arsenic: less than 6 μg  mercury: less than 2 μg,  copper: less than 150 μg  (Maximum daily usage) | | not detected |
| **3. Chromatographic fingerprinting** | | | | |
| consistent with the chromatographic retention time of reference standards | | | | qualified |
| Similarity no less than 0.85 | | | | 0.95 |
| **4. Content** | | | | |
| Total phenolic acids | calculated as danshensu sodium salt (C_9_H_9_O_5_Na): 4.0-12.0 mg/mL | | | 8.2 mg/mL |
| Danshensu sodium salt | calculated as danshensu sodium salt (C_9_H_9_O_5_Na): 0.60 mg/mL | | | 0.97 mg/mL |
| Protocatechuic aldehyde | calculated as rotocatechuic aldehyde (C_7_H_6_O_3_): 0.20 mg/mL | | | 0.30 mg/mL |
| Rosmarinic acid | calculated as Rosmarinic acid (C_18_H_16_O_8_): 0.10 mg/mL | | | 0.37 mg/mL |
| Salvianolic acid B | calculated as Salvianolic acid B (C_9_H_9_O_5_Na): 0.10 mg/mL | | | 0.48 mg/mL |
| **Conclusion:** Analytic samples meet the quality criteria of CFDA standard (No. WS3-B-3766-98-2011). | | | | |

Quality officer: Fei Xia Reviewing officer: Tian Tian
